# Supplementary material for: ATR inhibition facilitates targeting of leukemia dependence on convergent nucleotide biosynthetic pathways
Source: Nat Commun. 2017 Aug 14;8:241. doi: 10.1038/s41467-017-00221-3 (PMC5556071; doi:10.1038/s41467-017-00221-3)
Supplement: Supplementary file 1 — Supplementary Information [file 41467_2017_221_MOESM1_ESM.pdf]

File Name: Supplementary Information
Description: Supplementary Figures, Supplementary Methods and Supplementary References

File Name: Supplementary Data 1
Description: Percent glucose labeling and relative level for the identified metabolites in CEM cells treated as indicated for 12 h following release from G1 arrest. Results are from two combined independent LC-MS analysis each with triplicate wells.

File Name: Supplementary Data 2
Description: Protein expression levels normalized to control (NT) in CEM cells treated as indicated for 12 h following release from G1 arrest. Results are from three to four independent nLC-MS/MS analysis.

File Name: Supplementary Data 3
Description: Protein expression levels normalized to control (NT) in asynchronous CEM cells treated with 1 μM VE-822 for 12 h. Results are from three independent nLC-MS/MS analysis.

File Name: Supplementary Data 4
Description: Heat map of the complete % labeling for the DNA LC-MS/MS-MRM analysis from Figure 4a.

File Name: Peer Review File
Description:

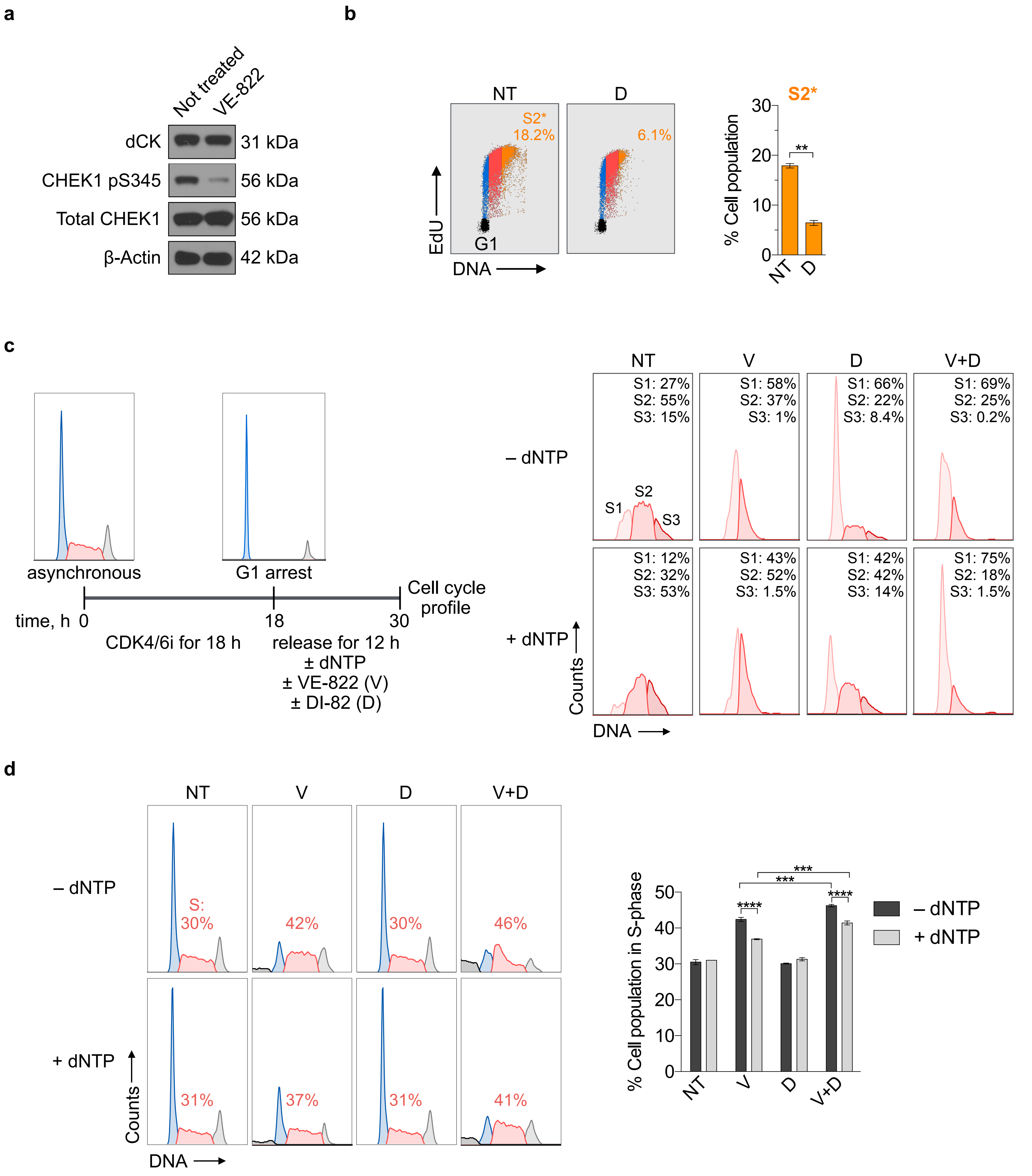

Supplementary Figure 1 | Constitutive ATR activation in CEM T-ALL cells and its role in cell cycle progression. (a) Representative pS345 CHEK1 immunoblots of CEM cells ± VE-822 (1 μM) for 24 h. **(b)** Flow cytometry analysis of EdU incorporation in CEM T-ALL cells treated with VE-822 (1 μM) and/or dCKi (DI-82,1 μM) for 12 h following release from G1 arrest. Bar graphs summarize the percentage of cell populations in S3 (late S-phase) 12 h (mean ± s.d., n = 2, one-way ANOVA, Bonferroni corrected). Plots are representative of two independent experiments. **(c)** Cell cycle analyses of synchronous CEM T-ALL cells treated with VE-822 (1 μM) and/or dCKi (DI-82,1 μM) in the presence or absence of 10 μM dNTPs for 12 h following release from G1 arrest. **(d)** Cell cycle analyses of asynchronous CEM T-ALL cells treated with VE-822 (1 μM) and/or dCKi (DI-82,1 μM) in the presence or absence of 10 μM dNTPs for 12 h. Bar graph summarize the percentage of cell populations in S-phase at 12 h (mean ± s.d., n = 2, one-way ANOVA, Bonferroni corrected).

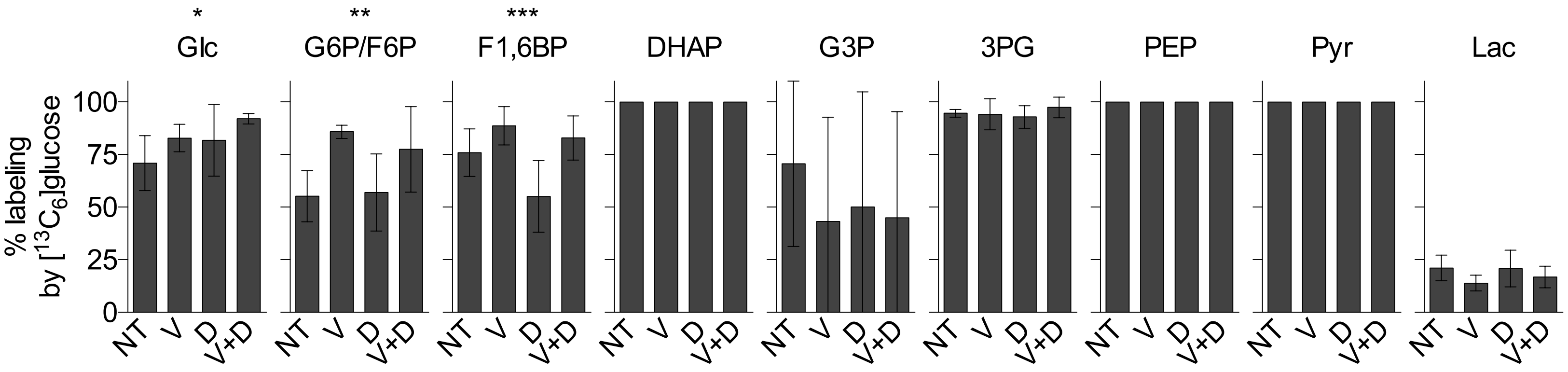

Supplementary Figure 2 | ATR inhibition minimally impacts glucose labeling in the glycolysis of CEM T-ALL cells. Percent glucose labeling of glycolytic metabolites (mean ± s.d., n = 6, one-way ANOVA).
Glc glucose; **G6P/F6P** glucose 6-phosphate/fructose 6-phosphate; **F1,6BP** fructose 1,6-bisphosphate; **DHAP** dihydroacetone phosphate; **G3P** glyceraldehyde 3-phosphate; **3PG** 3-phosphoglycerate; **PEP** phosphoenolpyruvate; **Pyr** pyruvate; **Lac** lactate.
* $P < 0.05$; ** $P < 0.01$; *** $P < 0.001$.

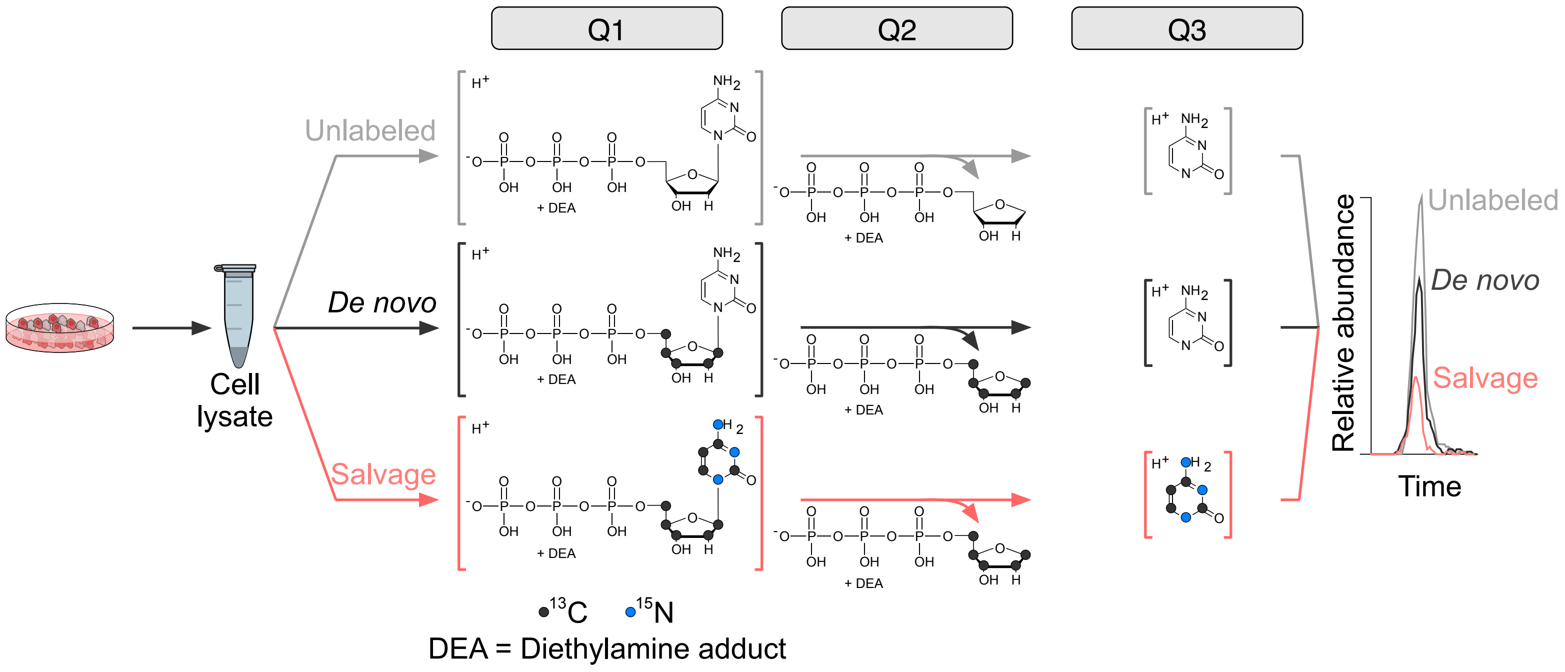

Supplementary Figure 3 | Workflow for the targeted mass spectrometric analysis used in Fig. 2 to measure dCTP pools. Optimally, nucleotides should be monitored in the positive mode to precisely measure the labeled atoms on the nucleobase and sugar moieties. To enable such measurements, we expanded on the work by Cohen and colleagues[1] who reported the use of ion pairing reagents. With the addition of diethylamine (DEA) in the mobile phase, nucleotides have their negative charge phosphate group masked by DEA and their nucleobase becomes protonated. Under these conditions, nucleotides with the DEA adduct were monitored in the positive mode as $[NTP{-}DEA{-}H]^+$ and the fragmentation of the adduct nucleotide ions resulted in protonated nucleobases.

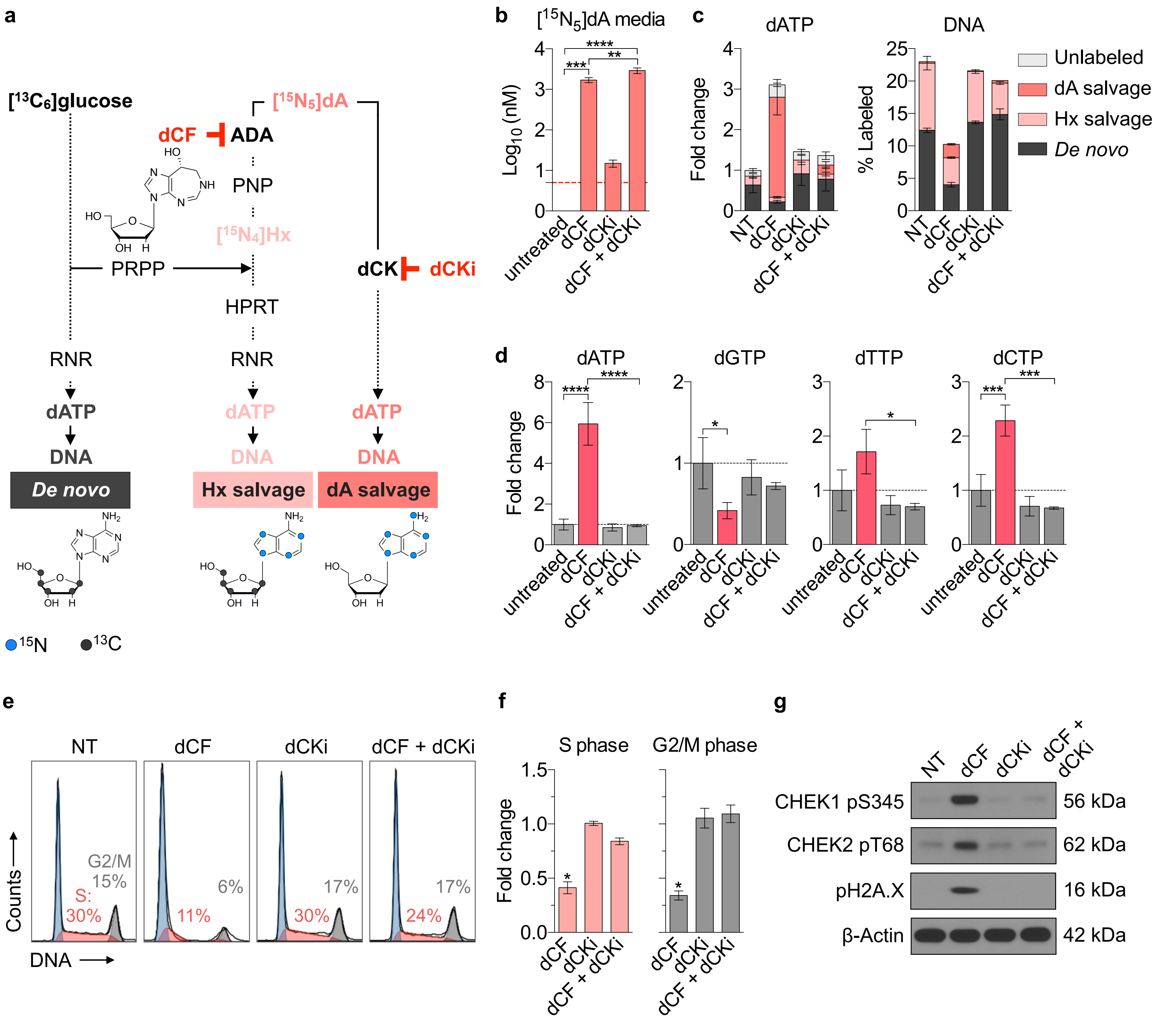

Supplementary Figure 4 | dCK-dependent dATP production from dA requires ADA inhibition (companion for Fig. 1). (a) Schematic representation and the ^{13}C and ^{15}N labeling pattern of the *de novo* and salvage dATP biosynthetic pathways. dA can be salvaged as a nucleobase through the actions of ADA, PNP, and HPRT or as an intact deoxyribonucleoside via dCK. The former salvage pathway is blocked by Pentostatin (dCF), a specific adenosine deaminase (ADA) inhibitor; the latter salvage pathway is blocked by DI-82, a dCK inhibitor (dCKi). **(b–d)** Jurkat cells were treated with dCF (10 μM) ± dCKi (1 μM) in culture media containing 11 mM [$^{13}C_6$]glucose and 5 μM [$^{15}N_5$]dA for 18 h. **(b)** [$^{15}N_5$]dA levels in the cell culture media 18 h after treatment initiation (mean ± s.d., n = 3, one-way ANOVA, Bonferroni corrected). **(c)** Biosynthetic routes to produce free dATP and dATP incorporated into newly replicated DNA 18 h after treatment initiation: *de novo* from [$^{13}C_6$]glucose via RNR, [$^{15}N_5$]dA salvage via dCK catalyzed phosphorylation, and HPRT mediated salvage of [$^{15}N_5$]hypoxanthine (Hx) produced from [$^{15}N_5$]dA following deamination, and glycosidic bond cleavage catalyzed by ADA and PNP, respectively (mean ± s.d., n = 3). **(d)** Fold changes in total dNTP levels 18 h after treatment initiation (mean ± s.d., n = 3, one-way ANOVA, Bonferroni corrected). **(e)** Cell cycle analyses of Jurkat cells treated as indicated for 24 h. **(f)** Quantification of cells in S (left panel) and G2/M (right panel) after the indicated treatments for 24 h (mean ± s.d., n = 2). **(g)** Representative immunoblots of CHEK1, CHEK2 and H2A.X phosphorylation; cells were treated as indicated for 24 h.
* $P < 0.05$; ** $P < 0.01$; *** $P < 0.001$; **** $P < 0.0001$.
dCF Pentostatin; **ADA** adenosine deaminase; **PNP** purine phosphorylase; **Hx** hypoxanthine; **HPRT** hypoxanthine phosphoribosyltransferase.

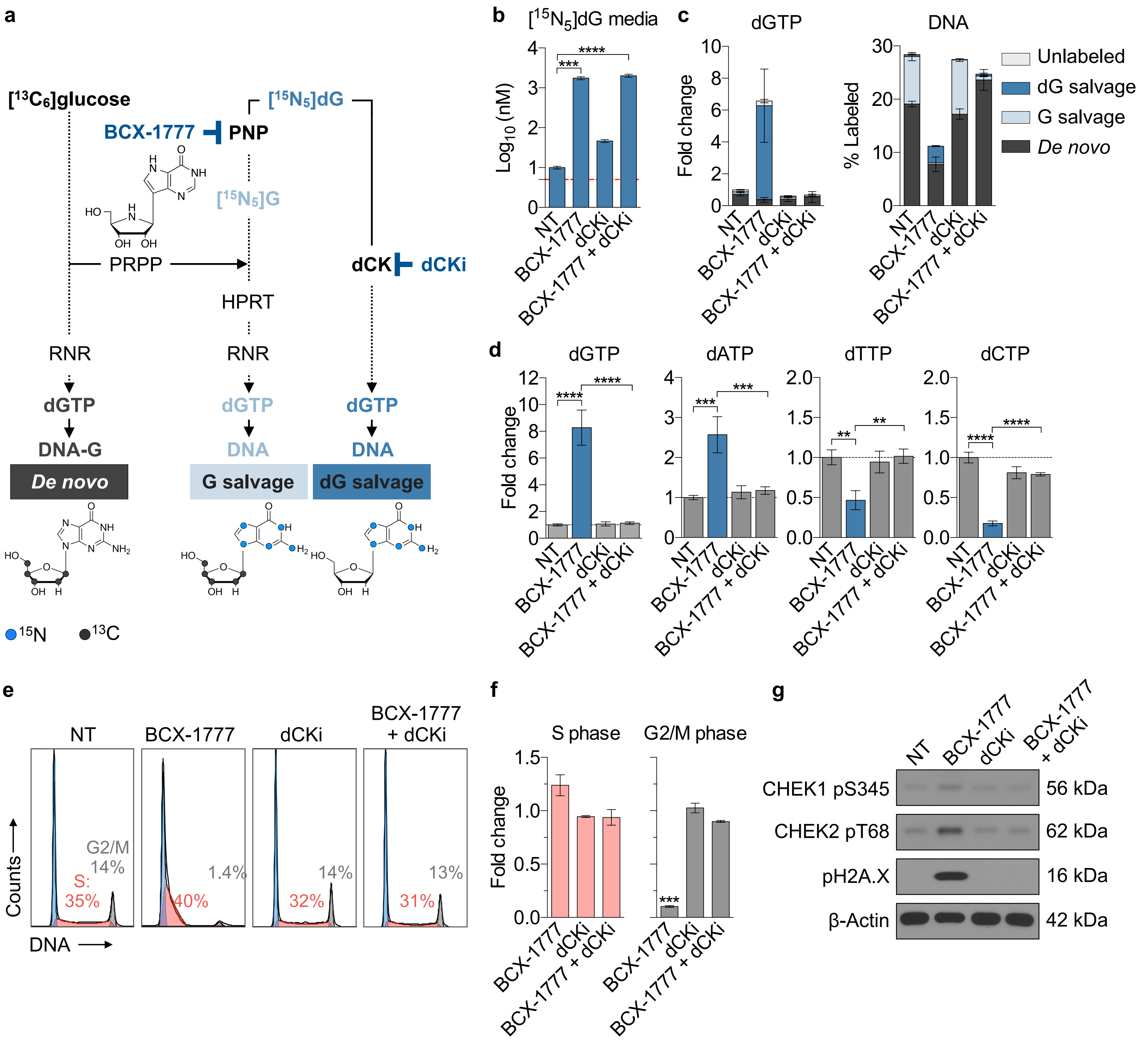

Supplementary Figure 5 | dCK-dependent production from dG requires PNP inhibition (companion for Fig. 1). (a) Schematic representation and the ^{13}C and ^{15}N labeling pattern of the *de novo* and salvage dGTP biosynthetic pathways. dG can be salvaged as a nucleobase through the actions of PNP and HPRT or as an intact deoxyribonucleoside via dCK. The former salvage pathway is blocked by BCX-1777, a specific inhibitor of PNP; the latter salvage pathway is blocked by DI-82, a dCK inhibitor (dCKi). **(b–d)** Jurkat cells were treated with BCX-1777 (100 nM) ± dCKi (1 μM) in culture media containing 11 mM [$^{13}C_6$]glucose and 5 μM [$^{15}N_5$]dA for 18 h. **(b)** [$^{15}N_5$]dG concentration in the cell culture media 18 h after treatment initiation (mean ± s.d., n = 3, one-way ANOVA, Bonferroni corrected). **(c)** Biosynthetic routes to produce free dGTP and dGTP incorporated into newly replicated DNA 18 h after treatment initiation: *de novo* from [$^{13}C_6$]glucose via RNR, [$^{15}N_5$]dG salvage via dCK catalyzed phosphorylation, and HPRT mediated salvage of [$^{15}N_5$]G produced from [$^{15}N_5$]dG following glycosidic bond cleavage catalyzed by PNP (mean ± s.d., n = 3). (**d**) Fold changes in total dNTP levels 18 h after treatment initiation (mean ± s.d., n = 3, one-way ANOVA, Bonferroni corrected). (**e**) Cell cycle analyses of Jurkat cells treated as indicated for 24 h. **(f)** Quantification of cells in the S (left panel) and G2/M (right panel) after the indicated treatments for 24 h (mean ± s.d., n = 2, one-way ANOVA, Bonferroni corrected). (**g**) Representative immunoblots of CHEK1, CHEK2 and H2A.X phosphorylation; cells were treated as indicated for 24 h
* $P < 0.05$; ** $P < 0.01$; *** $P < 0.001$; **** $P < 0.0001$.
PNP purine phosphorylase; **Hx** hypoxanthine; **HPRT** hypoxanthine phosphoribosyltransferase.

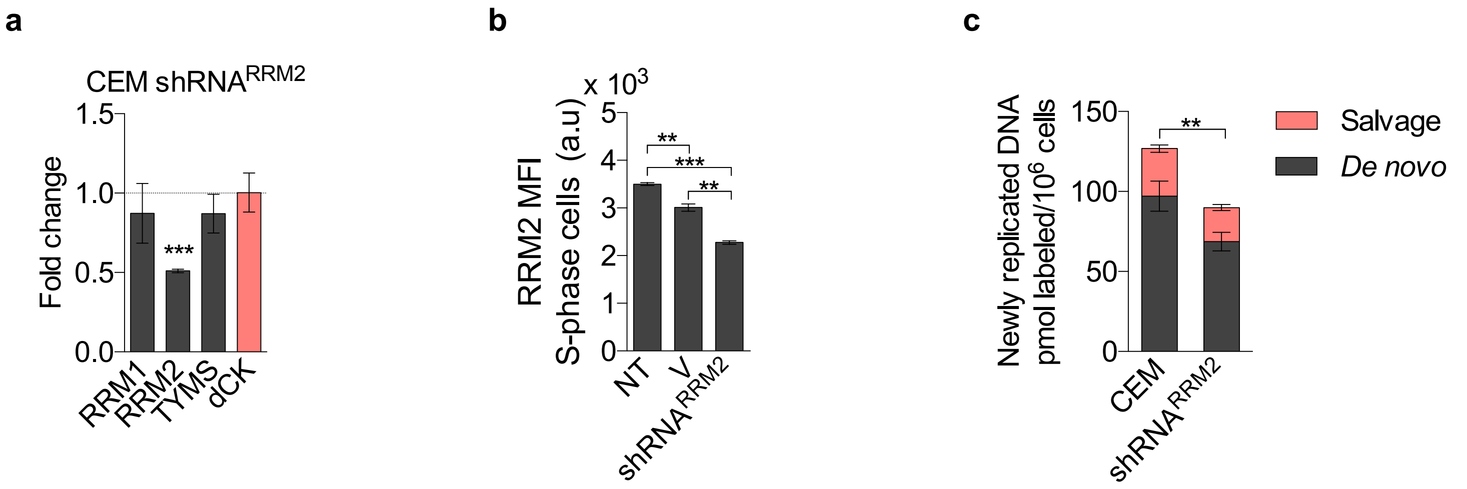

Supplementary Figure 6 | Effects of knocking down RRM2 using shRNA on RRM2 protein levels and RNR-produced dCTP incorporated into newly replicated DNA of CEM T-ALL cells. (a) Protein levels in CEM shRNA^{RRM2} cells as determined by nLC-MS/MS (mean ± s.d., n = 3, one sample t-test to assess if the mean of the protein level normalized to parental CEM is equal to one). **(b)** Mean fluorescence intensity (MFI) of RRM2 levels in S-phase parental CEM cells treated with 1 μM VE-822 for 12 h (V) and CEM shRNA^{RRM2} cells (mean ± s.d., n = 2, one-way ANOVA, Bonferroni corrected). **(c)** Contributions of the *de novo* and salvage pathways to dCTP incorporated into newly replicated DNA in parental CEM and CEM shRNA^{RRM2} cells (mean ± s.d., n = 3, two-way ANOVA, Bonferroni corrected, comparing *de novo* contribution between parental CEM and CEM shRNA^{RRM2}).
* $P < 0.05$; ** $P < 0.01$; *** $P < 0.001$; **** $P < 0.0001$.
RRM1 ribonucleotide reductase subunit 1; **RRM2** ribonucleotide reductase subunit 2; **TYMS** thymidylate synthase; **dCK** deoxycytidine kinase.

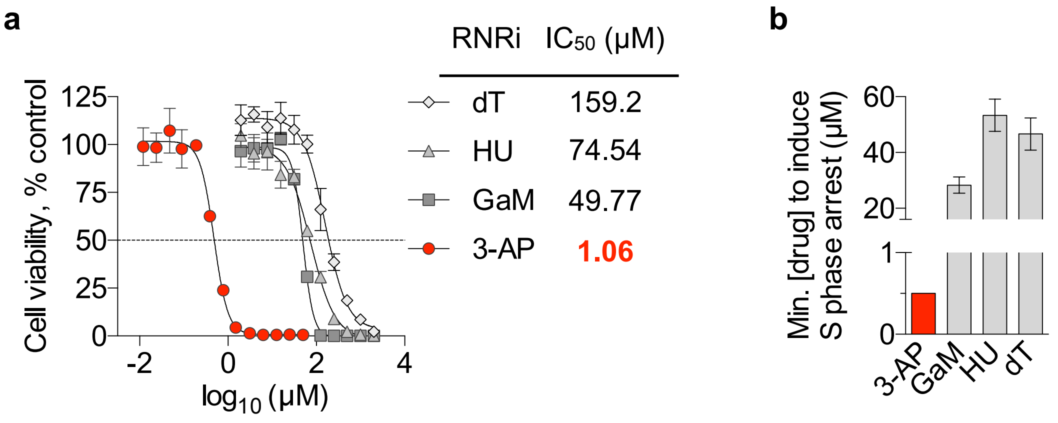

Supplementary Figure 7 | IC$_{50}$ values and minimal concentrations of RNR inhibitors required to cause S-phase arrest (see in Fig. 3b and main text for details). (a) IC$_{50}$ values of dT, HU, GaM, and 3-AP in CEM cells (CellTiter-Glo assay at 72 h, mean ± s.d., n = 3). **(b)** Minimal concentrations of the four RNR inhibitors required for the induction of S-phase arrest observed in Fig. 3b (see main text for details, mean ± s.d., n = 2, one-way ANOVA, $P < 0.0001$).
dT thymidine; **HU** hydroxyurea; **GaM** gallium maltolate.

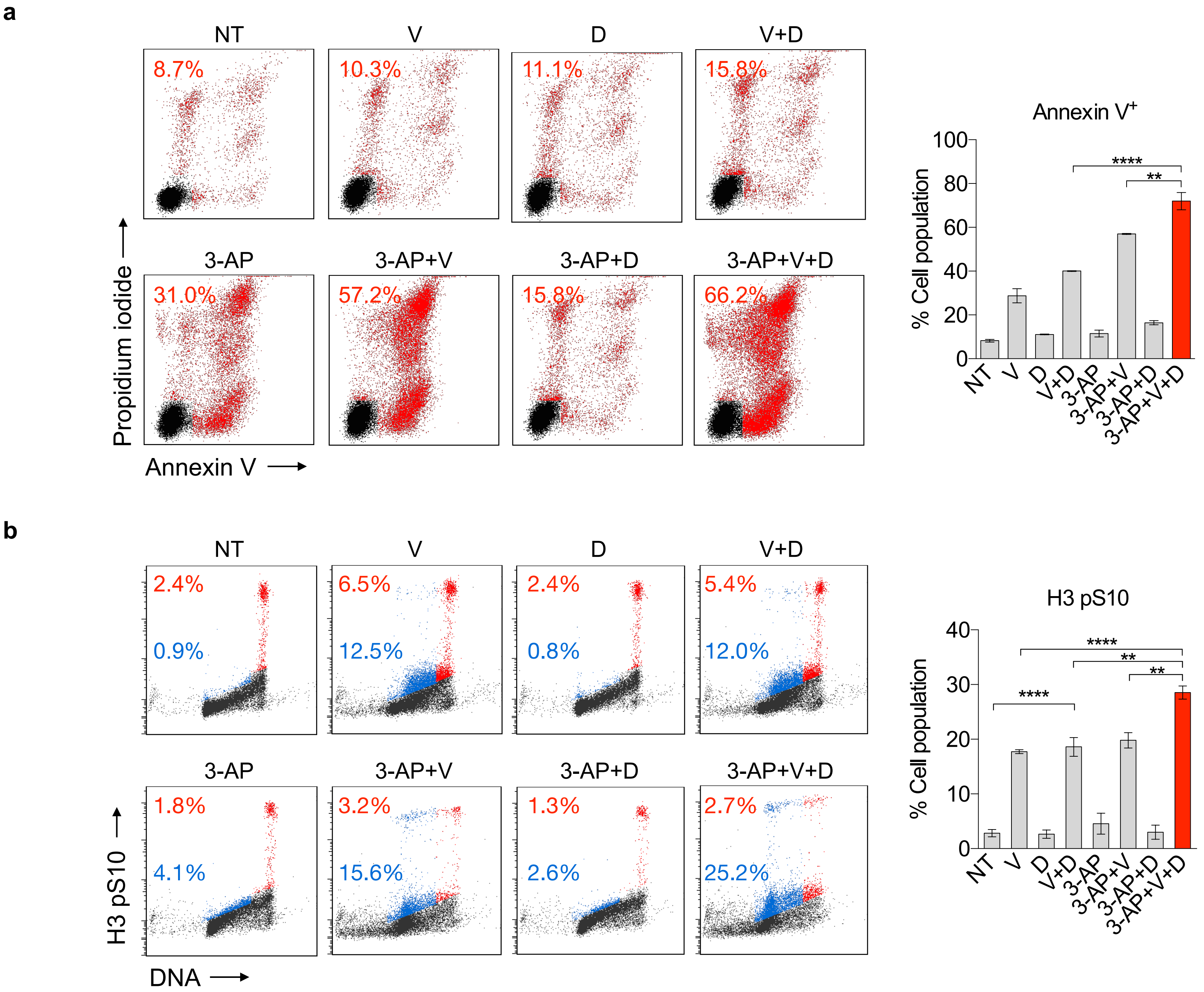

Supplementary Figure 8 | Analyses of apoptosis induction and premature mitotic entry in CEM cells treated with ATR, dCK and RNR inhibitors as indicated in Fig. 4. (a) Apoptosis induction in CEM cells treated as indicated (500 nM 3-AP, 1 μM VE-822, and 1 μM dCKi) for 24 h using flow cytometry for Annexin V and PI staining (mean ± s.d., n = 2, one-way ANOVA, Bonferroni corrected). **(b)** Phosphorylated H3 on serine 10 (pS10 H3) levels in CEM cells treated as indicated (500 nM 3-AP, 1 μM VE-822, and 1 μM dCKi) for 24 h using flow cytometry (mean ± s.d., n = 2, one-way ANOVA, Bonferroni corrected).
NT: not treated control, V: VE-822, D: dCKi.
* $P < 0.05$; ** $P < 0.01$; *** $P < 0.001$; **** $P < 0.0001$.

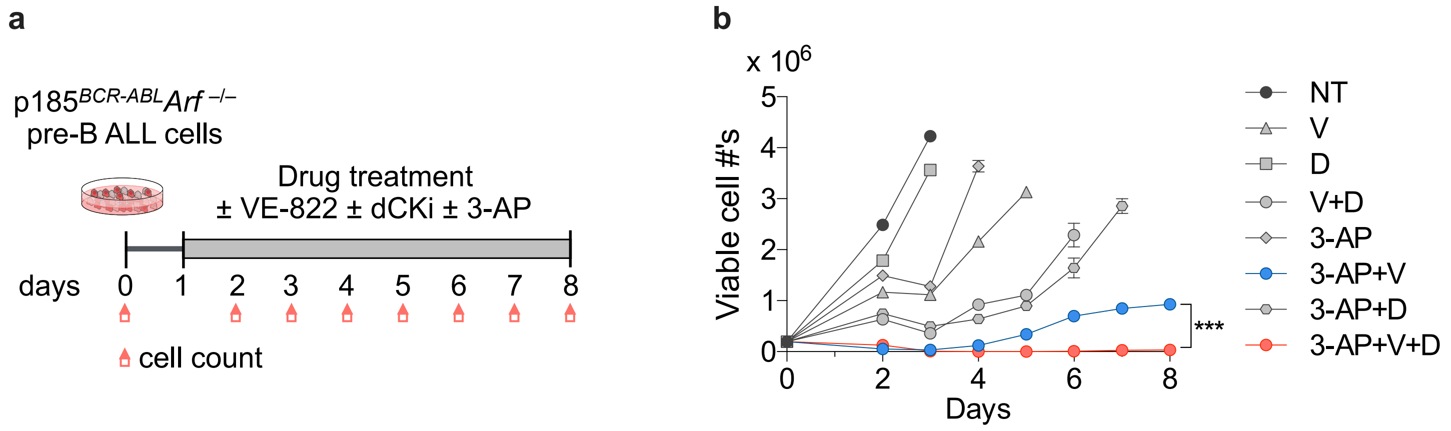

Supplementary Figure 9 | Optimal therapeutic efficacy against p185*BCR-ABLArf* –/– pre-B-ALL cells in culture requires combined inhibition of ATR, RNR and dCK (companion for Fig. 5). (a and b)** Treatment scheme **(a)** and growth curves **(b)** of p185$^{BCR\text{-}ABL}$*Arf*$^{-/-}$ pre-B-ALL treated as indicated (mean ± s.d., n = 2, unpaired two-tailed Student's t-test comparing 3-AP+V and 3-AP+V+D at day 8).
NT: not treated control, V: VE-822, D: dCKi.
*** *P* < 0.001.

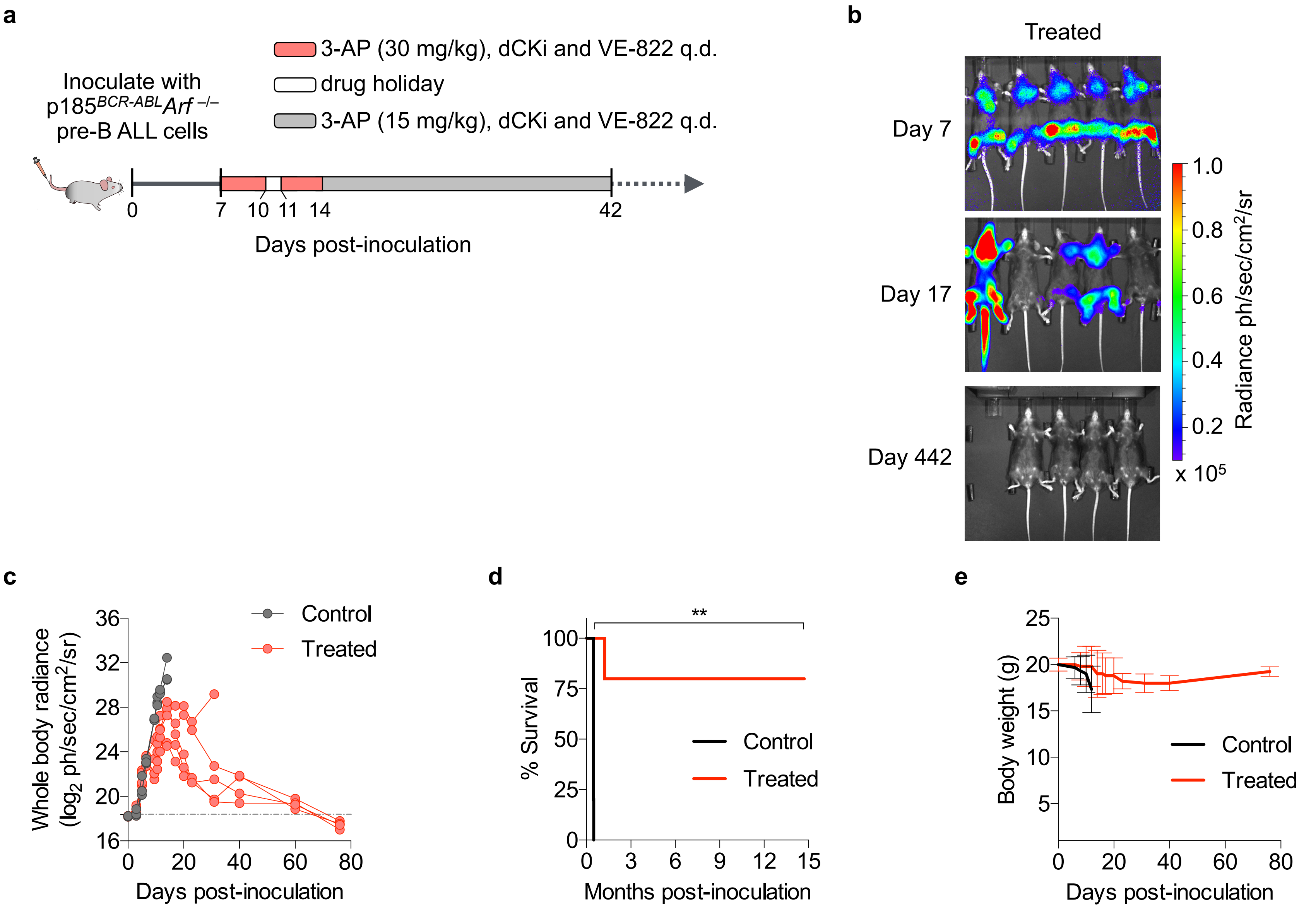

Supplementary Figure 10 | Co-targeting ATR, dCK and RNR is effective and well-tolerated in a systemic primary B-ALL model (companion for Fig. 6). (a, b, c) Schematic representation of the therapeutic regimen **(a)**, bioluminescence images **(b)**, and quantification of whole-body radiance **(c)** of leukemia bearing mice treated with a slight modification of the therapeutic regime shown in **Fig. 6b** (3-AP, 30 mg/kg q.d., treated, n = 5). **(d and e)** Kaplan-Meier survival analysis **(d)** and body weight measurements **(e)** of leukemia bearing mice treated with the combination therapy (treated, n = 5) or vehicle (control from **Fig. 6c**, n = 5). Median survival for the control group is 14 days after treatment initiation, whereas median survival for the treated group remains undefined (Mantel-Cox test).
** *P* < 0.01.

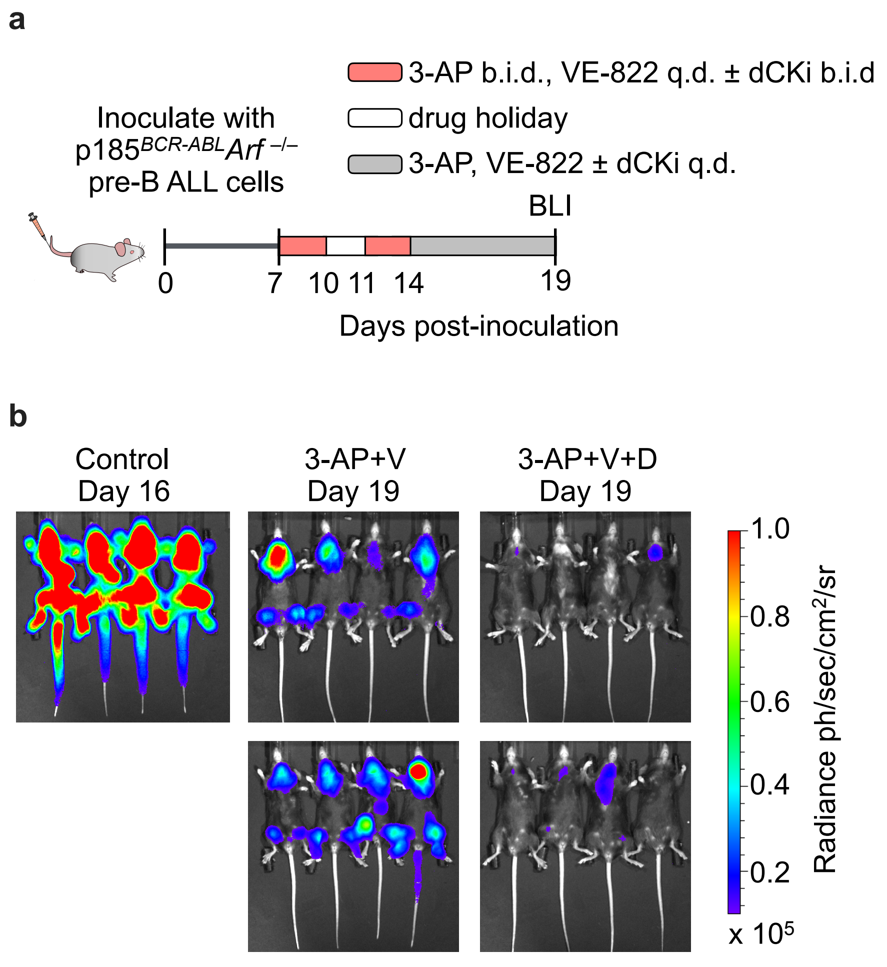

Supplementary Figure 11 | Optimal therapeutic efficacy against p185$^{BCR\text{-}ABL}$*Arf*$^{-/-}$ pre-B-ALL cells *in vivo* requires combined inhibition of ATR, RNR and dCK (companion for Fig. 6). (a) Schematic representation of the therapeutic regimen; **(b)** bioluminescence images **(c)**, and quantification of whole-body radiance of leukemia bearing mice treated with 15 mg/kg 3-AP + 40 mg/kg VE-822 (3-AP+V, n = 8), 15 mg/kg 3-AP + 40 mg/kg VE-822 + 50 mg/kg dCKi (3-AP+V+D, n = 8), and vehicle (control, n = 4). Drugs were formulated in PEG-200, Transcutol, Labrasol, and Tween-80 blended in a ratio of 5:3:1:1 and were administered orally. One-way ANOVA, Bonferroni corrected for the BLI comparisons among three groups at day 16 and unpaired two-tailed Student's t-test for the BLI comparison of 3-AP+V and 3-AP+V+D at day 19. Mice from control group were sacrificed before day 19 due to high leukemic burden.

* $P < 0.05$; ** $P < 0.01$; *** $P < 0.001$; **** $P < 0.0001$.

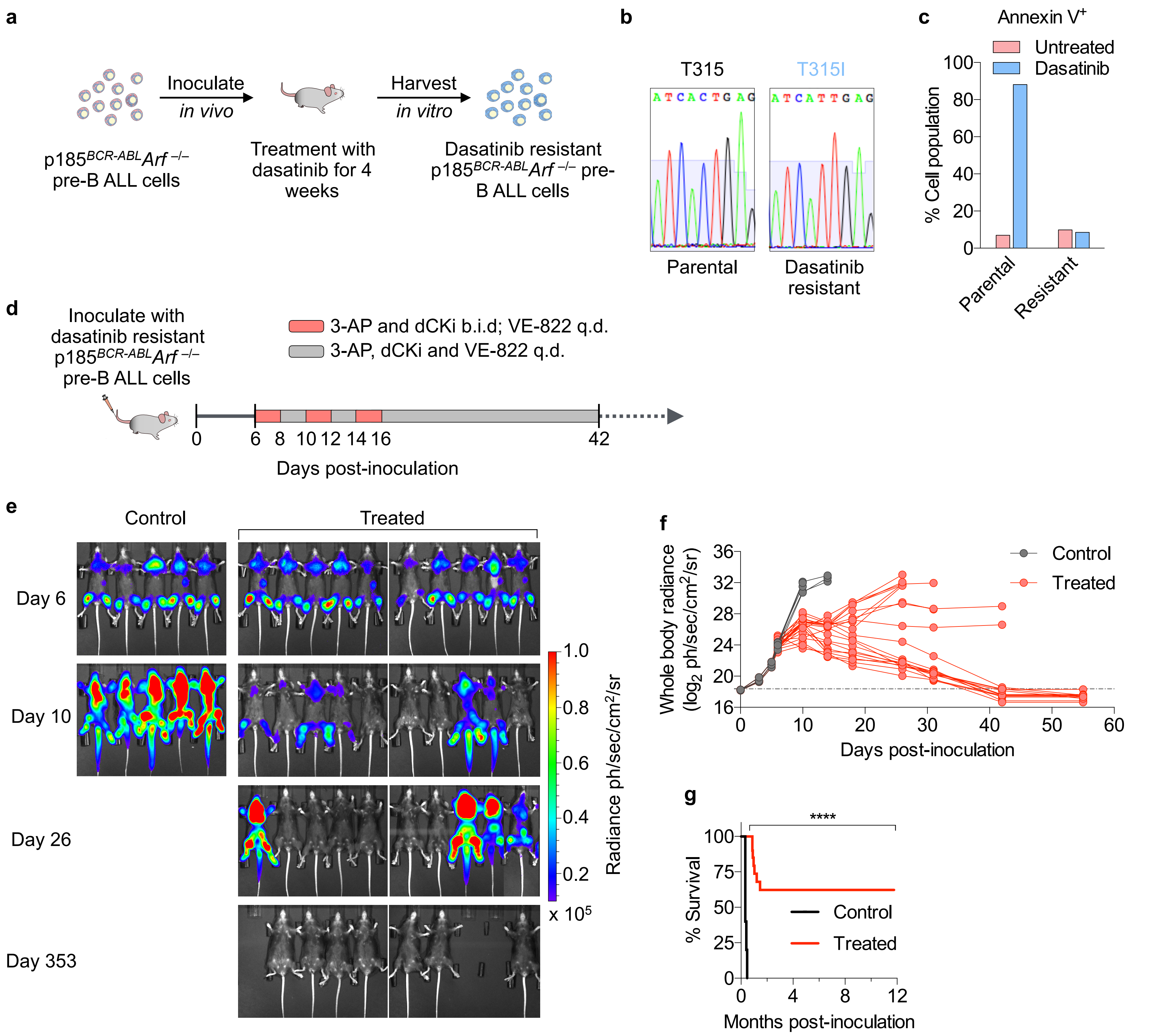

Supplementary Figure 12 | The combination therapy is efficacious against a dasatinib-resistant pre-B-ALL mouse model. (a) Generation of dasatinib-resistant p185$^{BCR\text{-}ABL}$*Arf*$^{-/-}$ pre-B-ALL cells. Leukemia bearing C57BL/6 mice, were treated with 10 mg/kg dasatinib q.d. (once/day) for 20 days. Upon presentation of resistance, leukemia cells were harvested from the bone marrow. **(b)** Sequencing of ABL kinase domain of dasatinib-resistant p185$^{BCR\text{-}ABL}$*Arf*$^{-/-}$ pre-B-ALL cells showed the T315I gate-keeper mutation. **(c)** Apoptosis induction in p185$^{BCR\text{-}ABL}$*Arf*$^{-/-}$ pre-B-ALL parental and resistant cells treated as indicated (dasatinib, 1 nM) for 24 h using flow cytometry for Annexin V and PI staining. **(d)** Doses and schedule of the combination treatment (3-AP, dCKi and VE-822) against dasatinib-resistant leukemia bearing mice. **(e and f)** Bioluminescence images **(e)** and quantification of whole body radiance **(f)** of dasatinib-resistant leukemia bearing mice treated with the combination therapy (treated, n = 20) or vehicle (control, n = 5) at indicated days after tumor inoculation. 10 representative mice are shown for the treated group. **(g)** Kaplan-Meier survival analysis of dasatinib-resistant leukemia bearing mice treated with the combination therapy (treated, n = 20) or vehicle (control, n = 5). Median survival for the control group is 14 days after treatment initiation, whereas median survival for the treated group remains undefined (Mantel-Cox test).
**** $P < 0.0001$.

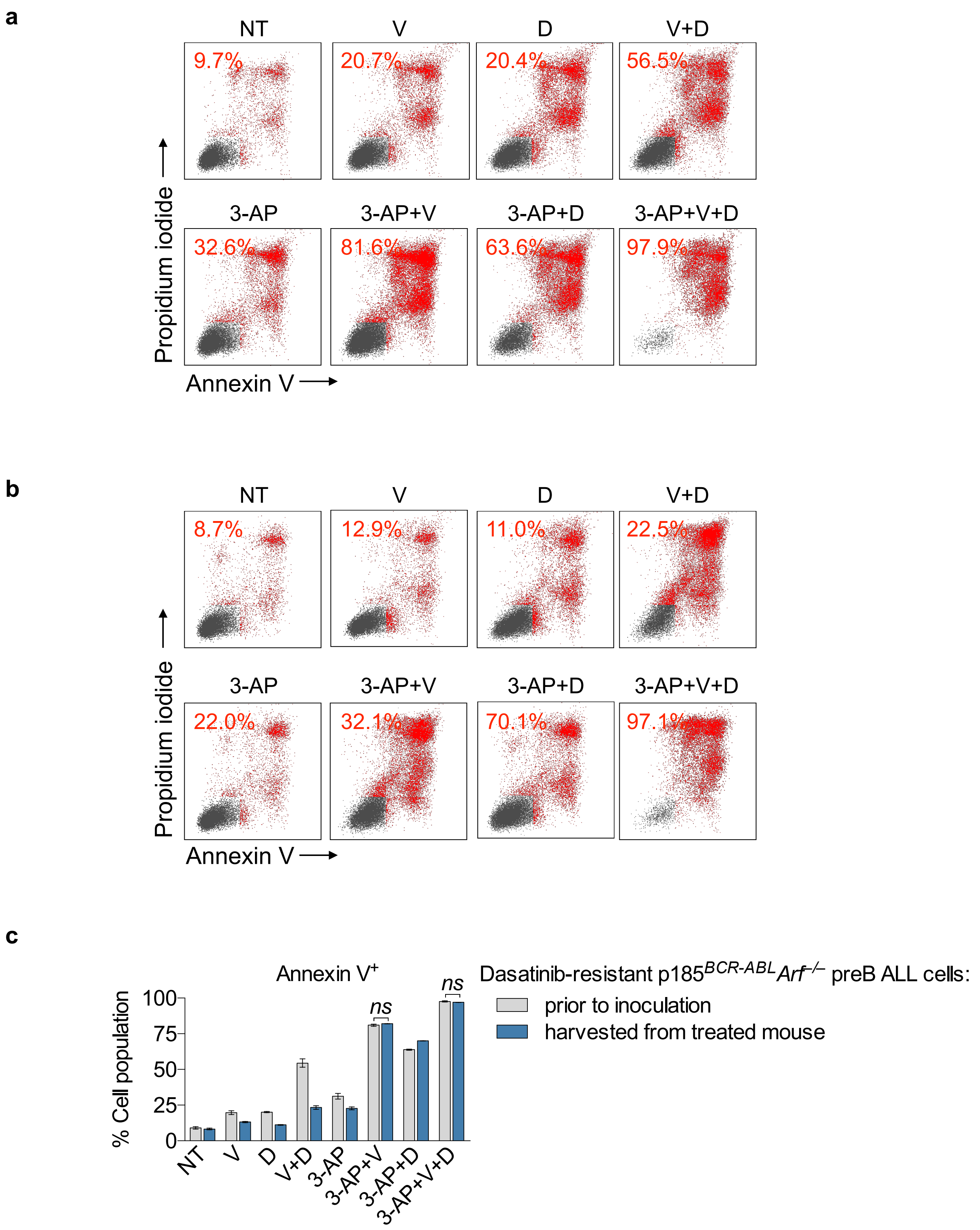

Supplementary Figure 13 | Dasatinib resistant pre-B-ALL cells recovered from a leukemic mouse which was treated with the triple combination therapy and eventually succumbed to disease retain sensitivity to the triple combination therapy. (a) Apoptosis induction of dasatinib-resistant p185$^{BCR\text{-}ABL}$*Arf*$^{-/-}$ pre-B-ALL cells, prior to inoculation in mice, treated as indicated (350 nM 3-AP, 100 nM VE-822, and 1 μM dCKi) for 72 h using flow cytometry for Annexin V and PI staining. **(b)** Apoptosis induction of dasatinib-resistant p185$^{BCR\text{-}ABL}$*Arf*$^{-/-}$ pre-B-ALL cells, harvested from bone marrow of a leukemic mouse treated with the triple combination therapy and eventually succumbed to disease from Supplementary Fig. 13, treated as indicated (350 nM 3-AP, 100 nM VE-822, and 1 μM dCKi) for 72 h using flow cytometry for Annexin V and PI staining. **(c)** Bar graph summarizes the percentage of Annexin V^{+} cell population from **(a)** and **(b)** (mean ± s.d., n = 2, one-way ANOVA, Bonferroni corrected).
ns not significant.

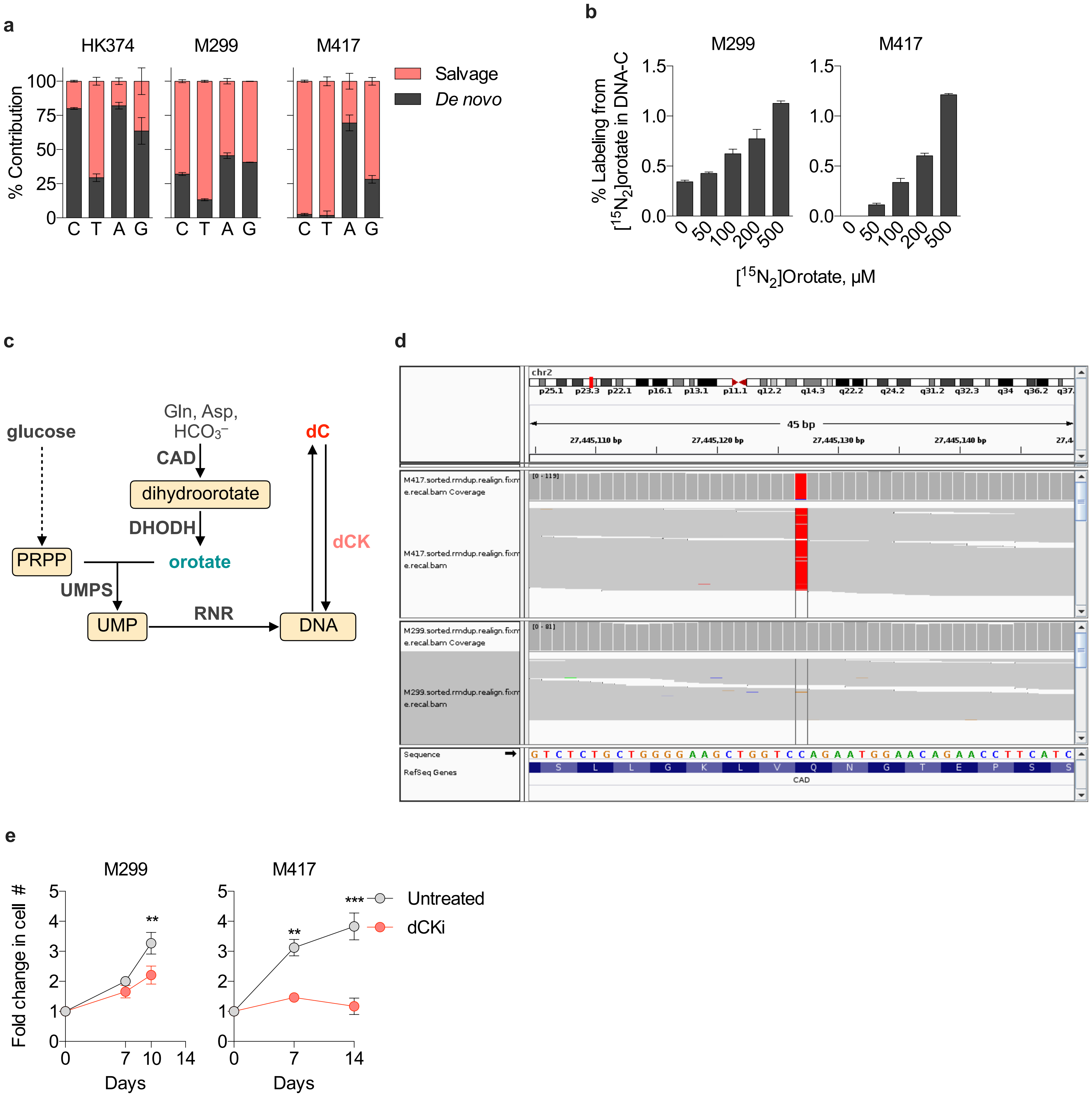

Supplementary Figure 14 | Nucleotide biosynthetic diversity in patient-derived samples. (a) Profiling the differential contributions of the *de novo* and salvage pathways to newly replicated DNA in patient-derived primary glioblastoma (HK374) and melanoma (M299 and M417) cells (mean ± s.d., n = 3). Cells were incubated in culture media containing 11 mM [$^{13}C_6$]glucose and 5 μM each of the following labeled nucleosides: [$^{13}C_9$,$^{15}N_3$]dC, [$^{13}C_{10}$,$^{15}N_2$]dT, [$^{13}C_{10}$,$^{15}N_5$]dA, [$^{15}N_5$]dG. **(b)** [$^{15}N_2$]orotate enrichment in dCTP incorporated into newly replicated DNA in M299 and M417 cells at 18 h (mean ± s.d., n = 3). Cells were incubated in culture media containing 11 mM [$^{13}C_6$]glucose with indicated varying concentration of [$^{15}N_2$]orotate. **(c)** Schematic representation of the *de novo* and salvage nucleotide pathway involving CAD, DHODH, and UMPS to newly replicated DNA. **(d)** IGV plot from exome sequencing of M417 showing homozygous CAD mutation (Q140*), present in 104 out of 108 reads. Results for M299 shown as negative reference. **(e)** Growth curves for M299 and M417 treated with vehicle or 1 μM dCKi (mean ± s.d., n = 2).
* $P < 0.05$; ** $P < 0.01$; *** $P < 0.001$; **** $P < 0.0001$.
CAD carbamoyl-phosphate synthetase 2, aspartate transcarbamylase, and dihydroorotase; **DHODH** dihydroorotate dehydrogenase; **UMPS** uridine monophosphate synthase; **PRPP** 5'-phosphoribosyl pyrophosphate; **UMP** uridine monophosphate.

Fig. 4C

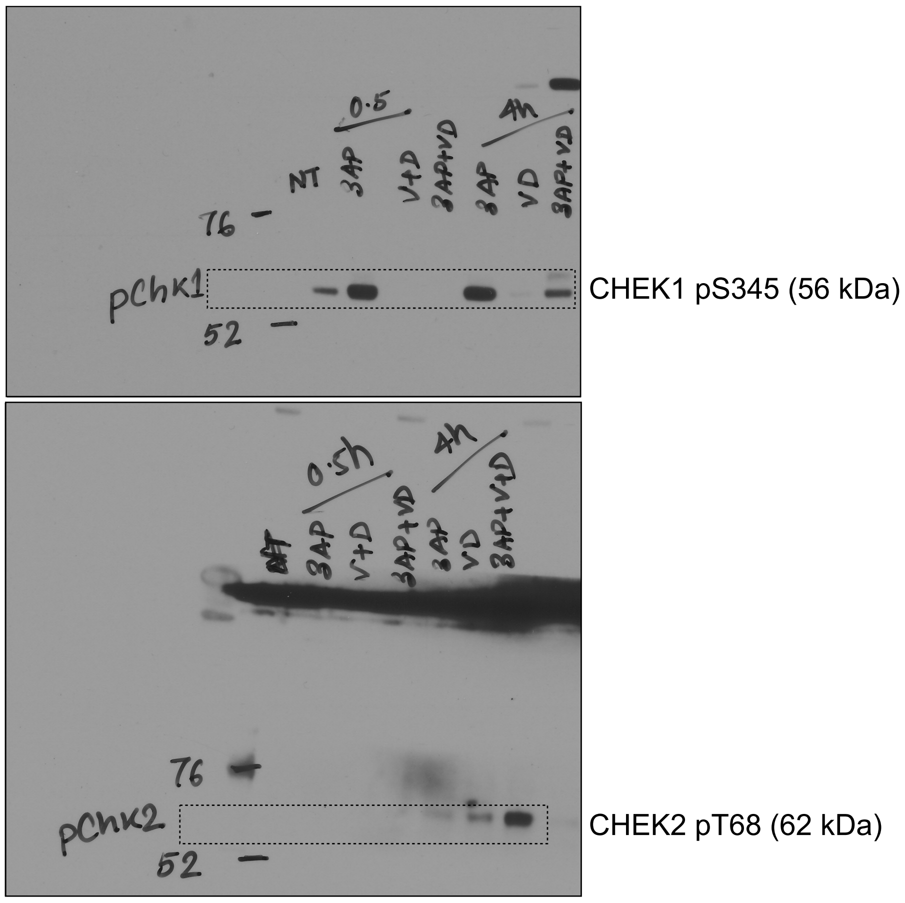

Fig. 4C

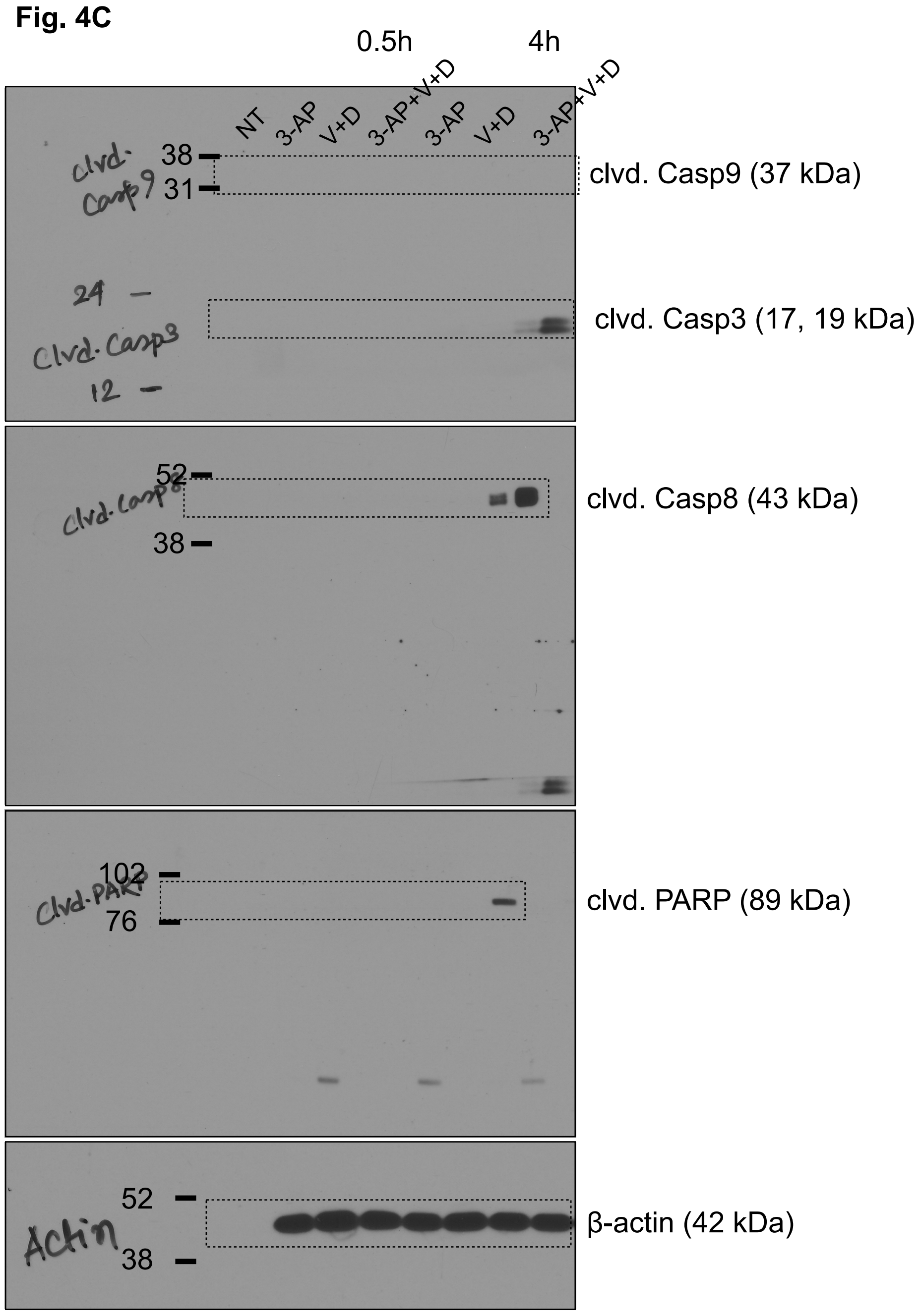

Fig. S1A

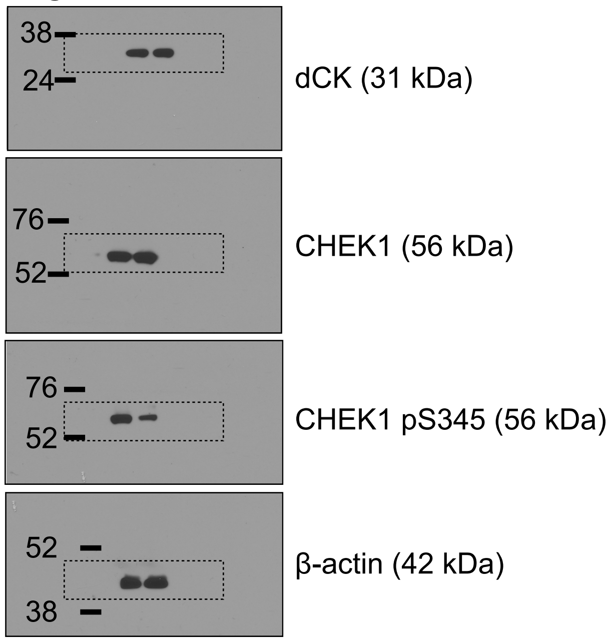

Fig. S4g and Fig. S5g

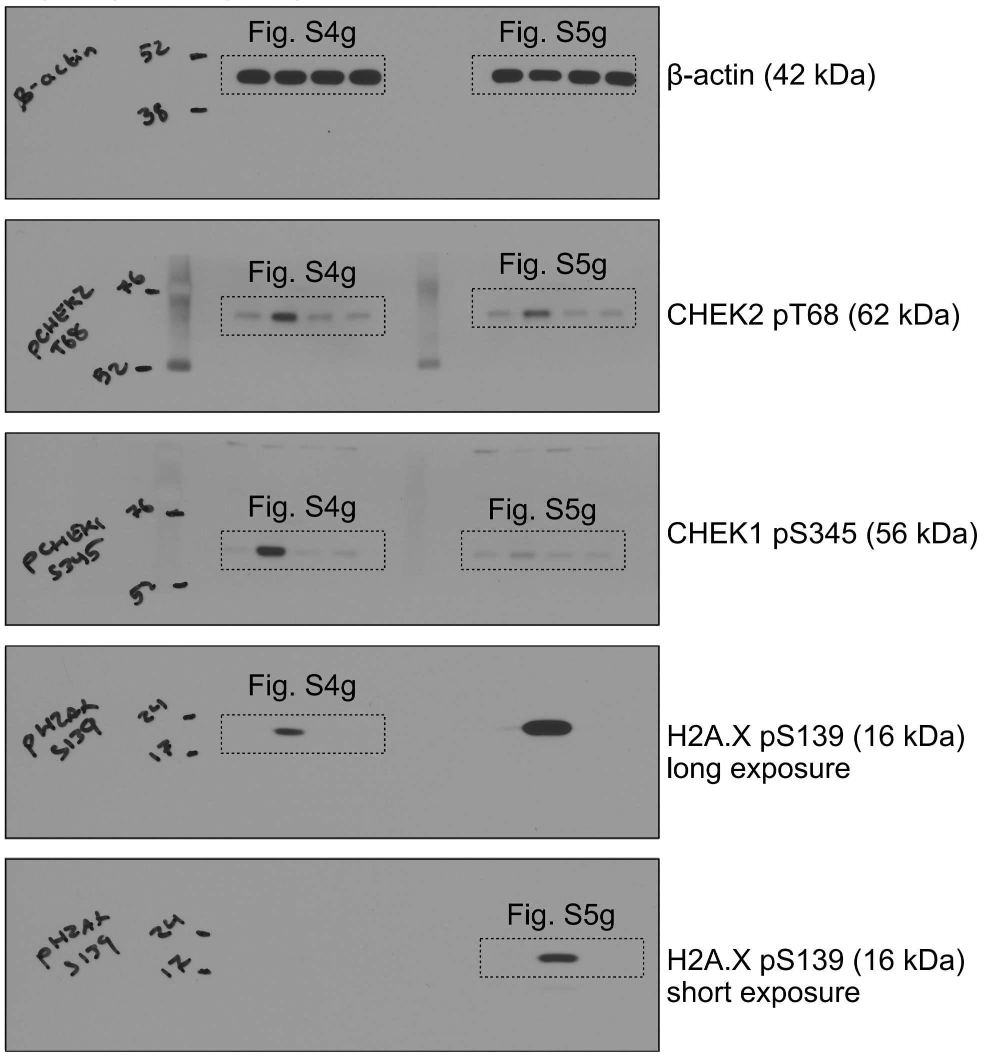

Supplementary Figure 15| Uncropped blots.

Supplementary Methods

Cell cycle synchronization. Cells were treated with a CDK4/6 inhibitor, Pablociclib or PD-0332991 (Selleckchem, S1116) for 18 h to synchronize them in the G1 phase. Subsequently, cells were washed twice with PBS containing 2% FBS (Omega Scientific) and released into fresh media.

Sequencing of dasatinib-resistant clones. Bone marrow cells were harvested from dasatinib treated mice with disease relapse during sacrifice and cultured in standard culture conditions. Genomic DNA was collected from resistant cell populations and a 2-step nested PCR strategy was utilized to amplify the human *ABL* kinase domain. PCR products were sequenced and assessed for the presence of T315I mutation.

Melanoma mutation assessment. Exome sequencing for M417 and M299 was performed at the UCLA Clinical Microarray Core on an Illumina HiSeq3000 using the SeqCap EZ Exome Enrichment Kit v3.0 (Roche). Reads were mapped to UCSC hg19 (bwa-mem), deduplicaed (PicardTools), and subjected to base quality score recalibration and realignment around known indels according to the Broad Institute Genome Analysis Toolkit (GATK) Best Practices v3.0. Variants in genes associated with nucleotide biosynthesis were identified using the GATK HaplotypeCaller, and annotated with Oncotator (http://portals.broadinstitute.org/oncotator/).

dNTP sample processing. 0.5 – 1 x 10^6 cells were collected into 1.5 mL microcentrifuge tubes and centrifuged (450 x g, 4 min, 4 ºC). The supernatant was carefully aspirated and the cells were washed twice with 1 mL of cold PBS, followed each time by centrifugation (450 x g, 4 min 4 ºC). The PBS were aspirated. Thereafter, the pellets were treated with 10 µL of 10% trifluoroacetic acid with internal standards (1 µM [$^{15}N_3$]dCMP and [$^{15}N_3$]dCTP, Silantes # 122303802 and # 120303802, respectively) vigorously vortexed for 30 s, and incubated on ice for 10 min. 40 µL of 500 mM ammonium acetate, pH = 9.3, with the same internal standard (1 µM [$^{15}N_3$]dCMP and [$^{15}N_3$]dCTP) was then added and the samples were vigorously vortexed again for 30 s. The samples were centrifuged (14,000 x g, 10 min, 4 ºC) to remove cell debris. The supernatants (~ 40 µL) were transferred into HPLC injector vials. Stock solutions (10 mM) of rCTP, rCDP, dCMP, dCDP, and dCTP (Sigma Aldrich) were prepared individually in water, and stored at –20 ºC before use to generate calibration standards. Calibration standards were prepared and mixed together in water with internal standards (1 µM [$^{15}N_3$]dCMP and [$^{15}N_3$]dCTP) to make working stock concentrations in 100 nM – 100 µM range. Calibration standards were diluted 10-fold into the same nucleotide extraction solution of 10% TFA/500 mM ammonium acetate (1:4, v/v) with internal standards (1 µM [$^{15}N_3$]dCMP and [$^{15}N_3$]dCTP) to give a final concentration in 10 nM – 10 µM. Nucleotide and calibration samples were processed together to minimize variation.

DNA sample processing. Genomic DNA from 0.5 – 1 x 10^4 cells was extracted using the Quick-gDNA MiniPrep kit (Zymo Research, D3021) and hydrolyzed to nucleosides using the DNA Degradase Plus kit (Zymo Research, E2021), following manufacturer-supplied instructions. In the final step of DNA extraction, 50 µL of

water was used to elute the DNA into 1.5 mL microcentrifuge tubes. A nuclease solution (5 µL; 10X buffer/DNA Degradase Plus™/water, 2.5/1/1.5, v/v/v) was added to 20 µL of the eluted genomic DNA in an HPLC injector vial. The samples were incubated overnight at 37 ºC.

Media samples processing. Culture media (20 µL) was collected at the indicated time points. Stock solutions (10 mM) of [U-$^{13}C_{10}$,$^{15}N_5$]dA, and [$^{15}N_3$]dC (Cambridge Isotope Laboratories) were prepared individually in dimethyl sulfoxide (DMSO), and stored at – 20 ºC before use as internal standards. The solutions were diluted to 20 nM in methanol to generate working solutions. Calibration standards were prepared by spiking working stock solutions of [U-$^{13}C_{10}$,$^{15}N_5$]dA and [U-$^{13}C_9$,$^{15}N_3$]dC with blank media to give concentrations in the 10 nM - 10 µM range. Each 20 µL calibration standard sample was mixed with 60 µL of internal standard solution, mixed for 30 s and centrifuged (15,000 x g, 10 min, 4ºC). After centrifugation, 60 µL of the supernatant was transferred into an HPLC injector vial for LC-MS/MS-MRM analysis. Media samples were processed similarly and in parallel to the calibration standard samples to minimize the experimental variability.

Targeted LC-MS/MS-MRM assays. For genomic DNA and media analysis, an aliquot of the hydrolyzed DNA or media samples (20 µL) were injected onto a porous graphitic carbon column (Thermo Fisher Scientific Hypercarb, 100 x 2.1 mm, 5 µm particle size) equilibrated in solvent A (water 0.1% formic acid, v/v) and eluted (200 µL/min) with an increasing concentration of solvent B (acetonitrile 0.1% formic acid, v/v) using min/%B/flow rates (µL/min) as follows: 0/0/200, 5/0/200, 10/15/200, 20/15/200, 21/40/200, 25/50/200, 26/100/700, 30/100/700, 31/0/700, 34/0/700, 35/0/200. For free nucleotide analysis, a modified version of the same previously reported method[1] was used in which each dNTP lysate sample (20 µL) was injected directly onto the Hypercarb column equilibrated in solvent C (5 mM hexylamine and 0.5% diethylamine, v/v, pH 10.0) and eluted (200 µL/min) with an increasing concentration of solvent D (acetonitrile/water, 50/50, v/v) at the following min/%D/flow rates (µL/min): 0/0/200, 5/0/200, 10/15/200, 20/15/200, 21/40/200, 25/50/200, 26/100/700, 30/100/700, 31/0/700, 34/0/700, 35/0/200. The effluent from the column was directed to the Agilent Jet Stream ion source connected to the triple quadrupole mass spectrometer (Agilent 6460) operating in the multiple reaction monitoring (MRM) mode using previously optimized settings. The peak areas for each nucleosides and nucleotides (precursor→fragment ion transitions) at predetermined retention times were recorded using the software supplied by the instrument manufacturer (Agilent MassHunter).

Quantification. The area for nucleotides measurements were obtained from extracted ion chromatograms of MRM ion transitions. These measurements were normalized from the spiked internal standards ([$^{15}N_3$]dCMP and [$^{15}N_3$]dCTP). A calibration curve was prepared to convert the normalized areas of nucleotides to absolute quantitation, pmol/10^6 cells. For DNA, the area for the hydrolyzed labeled nucleosides were obtained from extracted ion chromatograms of MRM ion transitions. These measurements were normalized to the total ion current at that retention time. To calculate the DNA labeled pmol/10^6 cells, the peak area ratio for a given biosynthetic pathway was multiplied by 1089.3, the dCTP pmol amount needed to replicate the entire genomic

DNA from 10^6 cells (assuming the size of the genomic DNA per cell to be 3.2 billion base pair with 41% GC content).

Non-targeted LC-MS metabolic assays. 1 x 10^6 cells were washed with ice-cold 150 mM ammonium acetate twice before adding 1 mL of ice-cold 80% methanol with 10 nM norvaline as an internal standard. After vigorous vortexing, samples were centrifuged at maximum speed, the aqueous layer was transferred to a glass vial and the metabolites were dried under vacuum. Metabolites were resuspended in 50 µL 70% acetonitrile (ACN) and 5 µL of this solution used for the mass spectrometer-based analysis. The analysis was performed on a Q Exactive (Thermo Scientific) in polarity-switching mode with positive voltage 4.0 kV and negative voltage 4.0 kV. The mass spectrometer was coupled to an UltiMate 3000RSLC (Thermo Scientific) UHPLC system. Mobile phase A was 5 mM ammonium acetate (NH_4AcO), pH 9.9, B was acetonitrile and the separation achieved on a Luna 3 mm NH2 100 A column (150 × 2.0 mm, Phenomenex). The flow was 200 µL/min, and the gradient ran from 15% A to 95% A in 18 min, followed by an isocratic step for 9 min and re-equilibration for 7 min. Metabolites were detected and quantified as area under the curve based on retention time and accurate mass (≤ 3 p.p.m.) using the TraceFinder 3.1 (Thermo Scientific). Relative amounts of metabolites between various conditions, as well as percentage of [$^{13}C_6$]glucose labelling, were calculated and corrected for naturally occurring ^{13}C abundance.

Proteomic analyzes. CCRF-CEM cells were treated ± with a CDK4/6 inhibitor, PD-0332991 (Selleckchem) for 18 h to arrest at G1 phase. Cells were then washed twice with PBS and released in fresh media with treatment at a density of 1 x 10^6 cells/mL. Cells were collected at 12 h, washed twice with ice-cold PBS, and lysed by trituration using 1 mL per 5 x 10^7 cells of 0.5% sodium deoxycholate, 12 mM sodium lauryl sarcosine, and 50 mM triethylammonium bicarbonate, pH 8.0. Lysates were heated at 95 ºC for 5 min and water bath sonicated at room temperature (RT) for 5 min. Bicinchoninic acid protein assay (Pierce) was performed to determine protein concentration. Disulfide bridges were reduced with 5 mM tris(2-carboxyethyl)phosphine (final concentration) at RT for 30 min with subsequent treatment with 10 mM iodoacetamide (final concentration) at RT in the dark for 30 min. Solutions were diluted 1:5 (v:v) with 50 mM triethylammonium bicarbonate. Proteins were cleaved with sequencing grade trypsin (Promega) at 1:100 (enzyme:protein) for 4 h at 37 ºC followed by a second aliquot of trypsin 1:100 (enzyme:protein) overnight at 37 ºC. Samples were acidified with 0.5% trifluoroacetic acid (final concentration), vortexed rapidly for 5 min, and centrifuged (16,000 x g for 5 min, RT) to pellet sodium deoxycholate. Supernatants were transferred to new microcentrifuge tubes and 20 µg of total peptide were desalted using C18 StageTips as previously described[2]. On-column dimethyl labeling using C18 StageTips was performed as previously described[3]. Briefly, StageTips were equilibrated with 20 µL of 250 mM 2-(N-morpholino)ethanesulfonic acid (MES) pH 5.5. Tryptic peptides were dimethyl labeled using 60 mM sodium cyanoborohydride, 0.4% formaldehyde, and 250 mM MES pH 5.5 for 10 min. Dimethyl labeled peptides were eluted from StageTips using 20 µL of 80% acetonitrile with 0.1% trifluoroacetic acid and lyophilized to dryness. Due to the nature of comparing 4 sample groups and being limited to 3 channels for stable isotope reductive amination labeling, two independent multiplexed experiments were run with each

independent experiment including the non-treated sample group as the bridge sample, in order to compare treated samples across the experiments. Therefore, one multiplexed experiment contained non-treated, VE-822 treated, and VE-822 + dCKi treated sample groups and the other multiplexed experiment contained non-treated and dCKi treated sample groups. Each multiplexed experiment was performed in triplicate. Labeled peptides were reconstituted with 2% acetonitrile and 0.1% formic acid (loading buffer). The light, medium, and heavy labeled peptides were mixed 1:1:1 (light:medium:heavy), diluted with loading buffer to a final peptide concentration of 0.2 µg/µL and 1 µg total peptide was analyzed using 180 min data-dependent nLC-MS/MS on Thermo Orbitrap XL as later discussed. Light, medium, and heavy labeled samples were mixed using the protein median ratios as normalization from the “trial” analysis. 48 µg of mixed light, medium, and heavy labeled peptides were sub-fractionated using strong cation exchange (SCX) StageTips as previously described[2]. Briefly, 8 fractions were made using 25, 35, 50, 70, 90, 150, 350, and 750 mM ammonium acetate in 30% acetonitrile and 0.5% acetic acid. Each SCX fraction was desalted using C18 StageTips, vacuum concentrated to 1 µL, and resuspended with 10 µL of loading buffer. 5 µL of each fraction was analyzed using 180 min data-dependent reverse-phase nLC-MS/MS on Thermo Orbitrap XL and Thermo QExactive Plus for synchronous and asynchronous cells, respectively, equipped with Eksigent Spark autosampler, Eksigent 2D nanoLC, and Phoenix ST Nimbus dual column source. Briefly, samples were loaded onto laser-pulled reverse-phase nanocapillary (150 µm I.D., 360 µm O.D. x 25 cm length) with C18 (300 Å, 3 µm particle size) (AcuTech Scientific) for 30 min with mobile phase A (2% acetonitrile and 0.1% formic acid) at 500 nL/min. Peptides were analyzed over 180 min non-linear gradient of 0-40% mobile phase B (98% acetonitrile and 0.1% formic acid) at 500 nL/min. Electrospray ionization and source parameters for Orbitrap XL were as follows: spray voltage of 2.2 kV, capillary temperature of 200^oC, capillary voltage at 35 V, and tube lens at 90 V. Data-dependent MS/MS for Orbitrap XL was operated using the following parameters: full MS from 400-1700 m/z with 60,000 resolution at 400 m/z and target ion count of 3 x 10^5 or fill time of 700 ms, and twelve MS/MS with charge-state screening excluding +1 and unassigned charge states, isolation width of 2.0 m/z, target ion count of 5,000 or fill time of 50 ms, CID collision energy of 35, and dynamic exclusion of 30 sec. For QExactive Plus, the electrospray ionization and source parameters were as follows: spray voltage of 1.6 kV, capillary temperature of 200^oC, and S-lens RF level of 50. Data-dependent MS/MS for QExactive Plus was operated using the following parameters: full MS from 400-1700 m/z with 70,000 resolution at 400 m/z and target ion count of 3 x 10^6 or fill time of 100 ms, and twenty MS/MS with charge-state screening excluding +1 and unassigned charge states, 17,500 resolution at 400 m/z, isolation width of 2.0 m/z, target ion count of 50,000 or fill time of 50 ms, HCD collision energy of 27, and dynamic exclusion of 30 sec. Raw data was searched against Uniprot human database using MaxQuant 1.5.3.30 with standard preset search parameters. Briefly, the search parameters were as follows: 3-plex dimethyl labeling to lysine and peptide N-terminus, trypsin cleavage allowing up to 2 missed cleavages, fixed modification of carbamidomethyl to cysteines, variable modifications of acetylation to protein N-terminus and methionine oxidation, 10 ppm mass error for full MS, 0.5 Da and 20 mmu mass errors for MS/MS for Orbitrap XL and QExactive Plus, respectively, score of 40 or greater for modified peptides, peptide match between run feature with 1.5 min time window, and 1% false-discovery rate (FDR) on peptide and protein identifications. To calculate the FDR for the proteomics data, the MS/MS spectra were searched

against both the Uniprot human FASTA database and a decoy database of the Uniprot human database which read from C-terminus to N-terminus; Percolator was used to filter the data at 1% FDR at both the peptide and protein level.

Phosphoproteomic analyses. CCRF-CEM cells were prepared same as the asynchronous CEM cells total protein digests above, except that a total of 7.5 mg total protein from 1 x 10^8 cells was collected per treatment condition for phosphoproteomic analysis. tC18 Sep-Pak cartridges (Waters) were used for peptide desalting and Pierce Quantitative Colorimetric Peptide Assay was performed prior to phosphopeptide enrichment. Hydrophilic interaction chromatography (HILIC) and immobilized metal affinity chromatography (IMAC) were performed same as previously described[4]. Data dependent nLC-MS/MS was performed on Thermo QExactive Plus same as above. Raw data was searched against Uniprot human database using MaxQuant 1.5.3.30 with the following search parameters: trypsin cleavage allowing up to 2 missed cleavages, fixed modification of carbamidomethyl to cysteines, variable modifications of acetylation to protein N-terminus, methionine oxidation, and phosphorylation to serine, threonine, and tyrosine, 10 ppm and 20 mmu mass errors for full MS and MS/MS, respectively, score of 40 or greater for modified peptides, and 1% false-discovery rate on peptide and protein identifications. Identified phosphopeptides were manually quantified by area-based extracted ion chromatograms of the monoisotopic peak.

Plasma collection and pharmacokinetic assays. Blood was collected in heparin-EDTA tubes by retro-orbital technique at time points 0.25, 1, 6, and 24 h time points from the first set of mice and 0.5, 4, and 12 h from the second set of mice. Blood samples were spun at 2000 x g for 15 min and the plasma supernatants were collected. All plasma samples were frozen down at –20 ºC before sample processing. The stock solutions of 3-AP, VE-822, DI-82, 3-AP analog (NSC 266749), VE-821 (Selleckchem), DI-39 were prepared individually by dissolving the appropriate amount of each drug in a known volume of dimethyl sulfoxide (DMSO) to a 10 mM concentration and were stored at –20ºC before use. The 3-AP analog, VE-821 and DI-39 (internal standards) were diluted to 200 nM in methanol to make the internal solution. The calibration standards were prepared by spiking working stock solutions of 3-AP, VE-822 and DI-82 in plasma from untreated mice to give 0.01-10 pmol/µL range. Each 20 µL calibration standard sample was mixed with 60 µL of internal solution (methanol with 200 nM internal standards) and vortexed for 30 s. Following centrifugation at 15,000 x g for 10 min, approximately 60 µL of sample was carefully transferred into HPLC injector vials for LC-MS/MS-MRM analysis. Plasma samples were processed the same way as the calibration standard samples. 20 µL samples were injected onto a reverse phase column, (Thermo Scientific Hypersil GOLD column 3.0 µm; 2.1 x 100 mm) equilibrated in water/formic acid, 100/0.1, and eluted (200 µL/min) with an increasing concentration of solvent B (acetonitrile/formic acid, 100/0.1, v/v: min/% acetonitrile; 0/0, 5/0, 15/60, 16/100, 19/100, 20/0, and 25/0). The effluent from the column was directed to the Agilent Jet Stream ion source connected to the triple quadrupole mass spectrometer (Agilent 6460) operating in the multiple reaction monitoring (MRM) mode using previously optimized settings. The following drug precursor→fragment ion transitions were used: 3-AP (196→179), DI-82 (511→369), VE-822 (464→533), VE-821 (369→276), 3AP-analog (199→182), DI-39

(525→383). The peak areas for each drug (precursor→fragment ion transitions) at predetermined retention times were recorded using the software supplied by the instrument manufacturer (Agilent MassHunter).

Cell cycle histograms. Cells were plated at a density of 0.5 x 10^6 cells/mL in respective media or drug treated media. Following 24 h incubation, cells were harvested and washed twice with PBS twice before staining with 0.5 ml of propidium iodide (Calbiochem, #P3566, 1 µg/mL) solution containing Ribonuclease A and 0.3% Triton-X 100. The samples were protected from light before acquisition by flow cytometry.

Cell cycle kinetics and cell cycle progression by intracellular detection of DNA-incorporated 5-ethynyl-2-deoxyuridine (EdU). CEM T-ALL cells were plated at a density of 0.5 x 10^6 cells/mL. Cells were pulsed with 10 µM EdU (Invitrogen) for 1 h, washed twice with PBS, and released in fresh media containing 5 µM deoxyribonucleosides, with and without drugs. Cells were collected at different time points following release in fresh media, and then fixed with 4% paraformaldehyde and permeabilized using saponin perm/wash reagent (Invitrogen). Cells were then stained with azide-Alexa Fluor 647 by Click reaction according to manufacturer's protocol (Invitrogen; Click-iT EdU Flow cytometry kit, #C10634). The total DNA content was assessed by staining the samples with FxCycle-Violet (Invitrogen, #F10347) at 1 µg/mL final concentration in PBS containing 2% FBS. To measure the cell cycle progression of a synchronous population of cells, cells were first arrested in the G1 phase following treatment with 1 µM Palbociclib for 18 h, then washed and released in fresh media supplemented with drugs. Before collecting and fixing the cells at different time points, the cells were pulse-labeled with 10 µM EdU for 1 h, and then labeled with azide-Alexa Fluor 647 (Invitrogen; Click-iT EdU Flow cytometry kit). Total DNA content was assessed by staining with FxCycle-Violet (Invitrogen) at 1 µg/mL final concentration in PBS containing 2% FBS.

pH2A.X staining. Cells were harvested, fixed and permeabilized with cytofix/cytoperm (BD biosciences, #554722) for 15 min on ice protected from light. Cells were washed, then resuspended in 100 µL 1X Perm/wash buffer (BD Sciences) and incubated for 15 min on ice. The cells were washed, then resuspended in 50 µL with phospho-Histone H2A.X (Ser139) antibody conjugated to fluorochrome FITC (EMD Millipore, #05-636, 1:800 dilutions in perm/wash) for 20 min in the dark at RT. Subsequently, cells were washed and stained with 0.5 mL of DAPI (Invitrogen, #D1306) for DNA content (1 µg/mL in 2% FBS in PBS) before data acquisition.

Measurement of ssDNA levels using the F7-26 monoclonal antibody. Cells were harvested and fixed with ice-cold methanol:PBS (6:1 v/v) for 24h. Staining with F7-26 monoclonal mouse antibody was performed according to manufacturer's instructions (EMD Millipore, #MAB3299). Fixed cells were resuspended in 250 µL of formamide and heated in a water bath at 75 ºC for 10 min. Cells were then allowed to equilibrate to room temperature and then washed with 2 mL of 1% non-fat dry milk in PBS for 15 min. Subsequently, cells were resuspended in 100 µL of anti-ssDNA Mab F7-26 (EMD Millipore, 1:10 in 5% FBS in PBS) and incubated for 45 min at room temperature. Cells were washed with PBS and stained with 100 µL of fluorescence-conjugated

goat anti-mouse IgM antibody (Santa Cruz Biotechnology, #sc-3768, 1:50) for 45 min at room temperature. Cells were then washed with PBS, stained with 0.5 mL of DAPI for DNA content (1 µg/mL in 2% FBS in PBS) before data acquisition.

Phospho-histone 3 staining. Cells were treated with drug combinations of three drugs. 24 h following treatment, cells were fixed and permeabilized with 1% PFA and 1X Perm/wash solution respectively. Following fixation and permeabilization, cells were stained intracellularly with pH3 Ser10 antibody conjugated to AF647 (Cell signaling, #12230). Cells were then washed with PBS, stained with 0.5 mL of DAPI for DNA content (1 µg/mL in 2% FBS in PBS) before data acquisition.

Annexin V staining. Cells were treated with the indicated treatments for 24 or 72 h, then collected and washed twice with 1 mL of FACS buffer (2% FBS in PBS). Induction of apoptosis and cell death were assayed by staining the cells with Annexin V-FITC and PI according to manufacturer's instructions (FITC Annexin V Apoptosis Detection Kit, BD Sciences, #556570).

FACS analysis. All flow cytometry data were acquired on five-laser LSRII cytometers (BD) for analysis, and analyzed using FlowJo software (Tree Star). The cell cycle durations were calculated using equations for multiple time-point measurements according to previously published methods (Terry and White, 2006).

Supplementary References

1. Cohen, S. et al. Simultaneous analysis of eight nucleoside triphosphates in cell lines by liquid chromatography coupled with tandem mass spectrometry. *J Chromatogr B Analyt Technol Biomed Life Sci* **877**, 3831-3840 (2009).
2. Rappsilber, J., Mann, M. & Ishihama, Y. Protocol for micro-purification, enrichment, pre-fractionation and storage of peptides for proteomics using StageTips. *Nat Protoc* **2**, 1896-1906 (2007).
3. Wilson-Grady, J. T., Haas, W. & Gygi, S. P. Quantitative comparison of the fasted and re-fed mouse liver phosphoproteomes using lower pH reductive dimethylation. *Methods* **61**, 277-286 (2013).
4. Zappacosta, F., Scott, G. F., Huddleston, M. J. & Annan, R. S. An optimized platform for hydrophilic interaction chromatography-immobilized metal affinity chromatography enables deep coverage of the rat liver phosphoproteome. *J Proteome Res* **14**, 997-1009 (2015).
